# Nicotine Dependence and Rates of Postoperative Complications in Achilles Tendon Repair

**DOI:** 10.1177/10711007231205293

**Published:** 2023-10-30

**Authors:** Sterling DeShazo, Sabrina M. Pescatore, Vinod K. Panchbhavi

**Affiliations:** 1 The University of Texas Medical Branch at Galveston, Galveston, TX, USA

**Keywords:** nicotine dependence, complications, Achilles tendon repair

## Abstract

**Background:**

Nicotine is a modifiable risk factor that is well demonstrated to cause deleterious effects on tendon healing and overall health. Individuals that have a dependence on nicotine may be at an elevated risk for numerous postoperative complications when compared to nondependent patients. The purpose of this study is to evaluate the complications postoperatively between nicotine- and non–nicotine-dependent Achilles tendon repairs.

**Methods:**

The global health network database, TriNetX, was used to access and analyze deidentified patient information. Two cohorts were identified for the purposes of this study. Cohort A was defined as patients who had an Achilles tendon repair (*Current Procedural Terminology* [*CPT*]: 27650 or *CPT*: 27654) and had a dependence to nicotine (*International Classification of Diseases, Tenth Revision*, code: F17). Cohort B was defined as patients who had an Achilles tendon repair but did not have a dependence to nicotine. Data were gathered from health care organizations from August 21, 2000, to August 21, 2023. All postoperative complications were analyzed between 1 and 90 days after the Achilles tendon repair.

**Results:**

A total of 2117 nicotine-dependent patients were matched with 18 102 non–nicotine-dependent patients. After propensity matching for age at event, ethnicity, race, sex, and type 2 diabetes mellitus, nicotine-dependent patients who underwent Achilles tendon repair experienced higher associated risk for numerous postoperative complications. When compared to nondependent patients, nicotine-dependent patients had increased risk for wound disruption (risk ratio [RR] 1.55, 95% CI 1.11-2.16) and infection following a procedure (RR 1.64, 95% CI 1.09-2.47) within 90 days post Achilles tendon repair.

**Conclusion:**

In this database study using propensity matching, nicotine-dependent patients who underwent Achilles tendon repair were correlated with an increased associated risk for wound disruption and infection after a procedure than their non–nicotine-dependent counterpart.

**Level of Evidence:**

Level III, retrospective cohort study.

## Introduction

Achilles tendon rupture (ATR) is a relatively common and serious injury most often observed in young adult, male athletes, or the elderly.^15^ This injury poses significant limitations to the patient, including reduced athletic performance, mobility, and if not treated may lead to chronic pain or disability. Although the Achilles tendon is the thickest, strongest tendon in the body, there has been a significant increase over the last few decades in ATRs. This is likely due to increased participation in sports activities, an overall aging population, and a rise in obesity.^12,14,26^

Restoring normal structure and function often requires repair and intensive rehabilitation due to the poor vascular perfusion of the midsection of the tendon.^8,21^ However, surgical repair has been associated with substantial postoperative complications and possible reoperation or potential revision.^23^ The most common postoperative ATR complications include sural nerve injury, infection, rerupture, deep vein thrombosis, and hypertrophic scars.^18^ With the overall high risk for the development of post ATR complications, it is vital to identify and mitigate these modifiable risk factors.

Conflicting evidence exists regarding the impact of smoking with complications in the ATR postoperative period. Previous studies have identified tobacco use and smoking as potential risk factors; however, results have demonstrated varied significance.^19,25^ Literature has identified the harmful effects of nicotine on tendon healing using rat-based models, which suggests potential implications for humans following ATR repair.^4,7^ However, there are limited studies that assess the effects of nicotine dependency on humans and postoperative ATR complications.

Given the high-risk of postsurgical complications in ATR repair patients, even in the absence of comorbidities, it is crucial to identify predisposing risk factors that may exacerbate postoperative complications. For this reason, our study aims to analyze the effect of nicotine dependence and its potential effect on patients undergoing ATR. To the authors’ knowledge, no study of this manner has been performed.

## Methods

We utilized TriNetX, the global health collaborative research platform, which provides access to deidentified retrospective electronic medical records. We searched the US collaborative network for a query to 59 health care organizations (HCOs) and more than 93 million patients. The database query for deidentified patient data was generated from TriNetX on August 21, 2023. The data in this study was gathered from HCOs from August 21, 2000, to August 21, 2023. Because this study used only deidentified patient records and did not involve the collection, use, or transmittal of individually identifiable data, this study was exempted from Institutional Review Board approval.

Two cohorts were evaluated for this study. Achilles tendon repair was defined as a primary open or percutaneous repair (*Current Procedural Terminology* [*CPT*]: 27650), or secondary repair with or without a graft (*CPT*: 27654). Cohort A was defined as patients who had an Achilles tendon repair and had a dependence to nicotine (*International Classification of Diseases, Tenth Revision* [*ICD-10*]: F17). Cohort B was defined as patients who had an Achilles tendon repair but did not have a dependence to nicotine. Nicotine dependence (*ICD-10*: F17) as defined by the *ICD-10* criteria denotes patients who have some form of mental dependence to some form of nicotine. This includes but is not limited to patients with either complicated or uncomplicated dependence to cigarettes (*ICD-10*: F17.21), chewing tobacco (*ICD-10*: F17.22), or other tobacco products (*ICD-10*: F17.29). Cohorts were propensity matched for demographic factors, such as age at event, ethnicity, race, sex, and type 2 diabetes mellitus.

The outcomes evaluated were wound disruption (*ICD-10*: T81.3), infection following a procedure (*ICD-10*: T81.4), sepsis (*ICD-10*: A41.9), deep vein thrombosis (*ICD-10*: I82.40), and pulmonary embolism (*ICD-10*: I26). All outcomes were evaluated between 1 and 90 days postprocedure. Data are reported in risk ratios (RRs), 95% CIs, and a risk comparison expressed as a *P* value.

All statistical analyses were performed within the online TriNetX search platform. The TriNetX platform was used to perform a 1:1 propensity score matching using logistic regression. Cohort characteristics were specifically selected for cohort matching purposes. Demographic characteristics selected for propensity matching were age at index, male, female, unknown sex, African American, Asian, White, unknown race, Hispanic or Latino origin, not of Hispanic or Latino origin, and unknown ethnicity.

## Results

There was a total of 14 198 patients who underwent primary open or percutaneous Achilles tendon repair and 7191 patients who underwent secondary Achilles tendon repair with or without a graft. After exclusions, there was a total of 2117 patients who were nicotine-dependent and 18 102 non–nicotine-dependent patients who underwent either primary or secondary Achilles tendon repair. Nicotine-dependent patients who underwent Achilles tendon repair were on average 47 years old with an SD of 13 years. The cohort was 58% males, 42% females, and 0% unknown. Most patients were White (63%), followed by African American (24%), and then unknown race (12%). Of the 18 102 (89.5%) non–nicotine-dependent repairs, the average age was 46 years, with an SD of 15 years. The cohort was composed of majority men (61%) and was predominantly White (65%), followed by African Americans (18%), unknown race (14%), and Asian (3%).

Prior to cohort propensity matching, patients in the nicotine dependence group were more likely to be African American (*P* < .0001), or not of Hispanic or Latino ethnicity (*P* < .0001). Patients in the non–nicotine-dependent group were more likely to be Asian (*P* < .0001), unknown race (*P* = .001), or unknown ethnicity (*P* < .0001) (Table 1). After matching, all cohort discrepancies in each demographic category were nonsignificant (*P* > .05) (Table 2).

**Table 1.** Cohort Demographics Prior to 1:1 Propensity Matching of Nicotine-Dependent (*ICD-10*: F17) (1) vs Non–Nicotine-Dependent (2) Patients.

Cohort	Demographic	Mean ± SD	Patients	% of Cohort	*P* Value	Standard Difference
1	Age at index	46.8 ± 13.4	2092	100.0	.020	0.056
2		46.0 ± 15.3	17 713	100.0		
1	Male		1219	58.3	.031	0.050
2			10 754	60.7		
1	Female		872	41.7	.034	0.049
2			6958	39.3		
1	White		1312	62.7	.057	0.044
2			11 481	64.8		
1	African American		499	23.9	<.001	0.154
2			3124	17.6		
1	Asian		29	1.4	<.001	0.120
2			563	3.2		
1	Unknown race		233	11.1	.001	0.076
2			2419	13.7		
1	Not Hispanic or Latino		1615	77.2	<.001	0.178
2			12 288	69.4		
1	Hispanic or Latino		117	5.6	.077	0.042
2			1169	6.6		
1	Unknown ethnicity		360	17.2	<.001	0.169
2			4256	24.0		
1	Type 2 diabetes		347	16.6	<.001	0.273
2			1373	7.8		

**Table 2.** Cohort Demographics After 1:1 Propensity Matching of Nicotine-Dependent (*ICD-10*: F17) (1) vs Non–Nicotine-Dependent (2) Patients.

Cohort	Demographic	Mean ± SD	Patients	% of Cohort	*P* Value	Standard Difference
1	Age at index	46.8 ± 13.4	2092	100.0	.973	0.001
2		46.8 ± 13.4	2092	100.0		
1	Male		1219	58.3	.950	0.002
2			1221	58.4		
1	Female		872	41.7	.975	0.001
2			871	41.6		
1	White		1312	62.7	.974	0.001
2			1311	62.7		
1	African American		499	23.9	.971	0.001
2			500	23.9		
1	Asian		29	1.4	.894	0.004
2			28	1.3		
1	Unknown race		233	11.1	.922	0.003
2			235	11.2		
1	Not Hispanic or Latino		1615	77.2	.971	0.001
2			1614	77.2		
1	Hispanic or Latino		117	5.6	.946	0.002
2			118	5.6		
1	Unknown ethnicity		360	17.2	>.99	<0.001
2			360	17.2		
1	Type 2 diabetes		347	16.6	>.99	<0.001
2			347	16.6		

**Figure 1.**
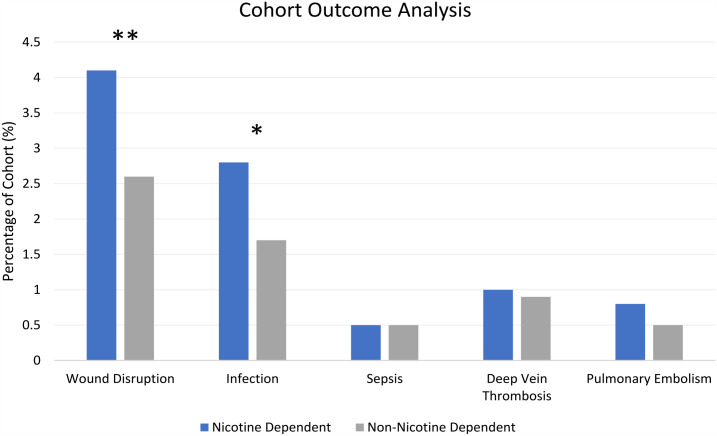
Cohort outcome assessment after propensity matching between nicotine-dependent patients (*ICD-10*: F17) (blue) vs non–nicotine-dependent patients (gray). One asterisk indicates a *P* value of <.05, whereas 2 asterisks indicate a *P* value of ≤.01. [See online article for color figure.]

Prior to propensity matching, nicotine-dependent patients who had an Achilles tendon repair were associated with a significantly higher risk for wound disruption, infection following a procedure, and sepsis when compared to their non–nicotine-dependent counterpart. After propensity matching, nicotine-dependent patients who had an Achilles tendon repair were associated with a significantly higher risk for wound disruption and infection following a procedure (Table 3, Figure 1).

**Table 3.** Outcome Evaluation After Propensity Matching of Nicotine-Dependent (*ICD-10*: F17) vs Nondependent Patients.

Outcome	Nicotine Dependent, n (%)	Non–Nicotine Dependent, n (%)	Risk Ratio (Dependent/Nondependent)	95% CI	*P* Value
Wound Disruptions	85 (4.1)	55 (2.6)	1.55	1.11-2.16	.01
Infection	59 (2.8)	36 (1.7)	1.64	1.09-2.47	.017
Sepsis	10 (0.5)	10 (0.5)	1.00	0.42-2.40	>.99
Deep Vein Thrombosis	21 (1.0)	19 (0.9)	1.11	0.60-2.05	.751
Pulmonary Embolism	16 (0.8)	10 (0.5)	1.60	0.73-3.52	.238

## Discussion

In this study, 2117 nicotine-dependent patients were matched with 18 102 non–nicotine-dependent patients who underwent Achilles tendon repair. We found that nicotine-dependent patients were at higher risk for operative complications, including wound disruption and infection following a procedure.

Smoking remains a well-characterized modifiable risk factor that has deleterious effects on tendon rupture rates and healing.^1,11,27^ Nicotine replacement therapy (NRT), such as electronic cigarettes, has been shown to be an alternative to cigarettes; however, further characterization on the impact of nicotine is required.^2,13^ It is hypothesized that nicotine stimulates catecholamines, like epinephrine, which results in peripheral vasoconstriction, which ultimately negatively affects tissue perfusion.^22^ Viable oxygenation and tissue perfusion are prerequisites to optimal tissue healing; thus, nicotine may negatively affect soft tissue healing.^16,24^

Nicotine has also been demonstrated to have deleterious effects on oxidative stress as well as significantly increase production of inflammatory markers, such as C-reactive protein, soluble intercellular adhesion molecule, and the danger signal machinery high-mobility group box 1 (HMGB1).^3,5,9^ This augmented state of inflammation may play a role in increased rates of perioperative infection and pneumonia.^10^

Current smoking cessation guidelines strongly advocate for smoking cessation before 4 weeks preoperatively, however, no difference in complications occurs if within the 4-week window.^6,20^ To our knowledge, no such guidelines have been established for nicotine-related products; furthermore, many guidelines advocate for NRT to aid patients in perioperative smoking cessation. There is evidence to suggest that NRT is effective in reducing many surgical complications, but further investigation of the efficacy of cessation is required.^17^

Although we think that this study possesses a valuable characterization on the impact of nicotine dependence on Achilles tendon repair complications, it does possess a number of different limitations. First, our data are sourced from a large database that heavily relies on administrative coding; thus, misclassification and residual confounding cannot be avoided. Moreover, the patient population included within the TriNetX system relies solely on participating HCOs and the quality of their reporting. Patients who receive care outside of participating HCOs would not be available for inclusion within this analysis. Second, studies with a retrospective, observational design inherently limit their generalizability to a larger population. For example, we do not know with any specificity the type of repairs that were done in this population (eg, open, mini-open, percutaneous) and whether this can impact rates of wound complications or early postoperative infections. Third, even though we controlled for a number of confounding variables, our analysis control is limited and may not completely account for all patient characteristics. Lastly, our data revolve around the *ICD-10* code for nicotine dependence, which may be used differently from physician to physician as it lacks specific, standardized definitions regarding dependence. For example, individual patient intensity of nicotine is not specified—so dose effect nor method of nicotine administration could not be investigated.

## Conclusion

In our retrospective observational propensity-matched cohort study, we found that nicotine-dependent patients who underwent Achilles tendon repair were associated with increased associated risk for operative complications including wound disruption and infection following a procedure within a 90-day postoperative period. Furthermore, future studies should seek to isolate the specific impact of nicotine from other confounding variables and whether preoperative cessation can impact incidence of complications within the acute postoperative period after surgical Achilles tendon repair.

## Supplemental Material

sj-pdf-1-fai-10.1177_10711007231205293 – Supplemental material for Nicotine Dependence and Rates of Postoperative Complications in Achilles Tendon Repair Click here for additional data file. Supplemental material, sj-pdf-1-fai-10.1177_10711007231205293 for Nicotine Dependence and Rates of Postoperative Complications in Achilles Tendon Repair by Sterling DeShazo, Sabrina M. Pescatore and Vinod K. Panchbhavi in Foot & Ankle International
